# Psychological capital mediates the mindfulness-creativity link: the perspective of positive psychology

**DOI:** 10.3389/fpsyg.2024.1389909

**Published:** 2024-08-29

**Authors:** Wu-jing He

**Affiliations:** The Education University of Hong Kong, Hong Kong, Hong Kong SAR, China

**Keywords:** creativity, mediation analysis, dispositional mindfulness, positive psychology, psychological capital

## Abstract

The positive mindfulness-creativity link has been widely documented; however, its underlying psychological mechanisms remain less understood. From the perspective of positive psychology, this study examined the mediating effect of psychological capital (PsyCap) on the effect of dispositional mindfulness on creative functioning. A total of 894 Chinese secondary school students in Hong Kong (50.8% female; *M*_age_ = 15.5 years) completed the study. A cross-sectional design was used, in which context PsyCap and dispositional mindfulness were assessed by the Chinese version of the revised Compound PsyCap Scale (CPC-12R) and the Mindful Attention Awareness Scale (MAAS), respectively. Moreover, by adopting the multiple-measurement approach to creativity, three commonly used creativity tests (i.e., the Wallach-Kogan Creativity Test/WKCT, the Test for Creative Thinking–Drawing Production/TCT–DP, and the Creative Problem-Solving Test/CPST) were applied to capture three aspects of creativity (i.e., divergent thinking, creative combination, and creative problem solving). The results suggest that (1) PsyCap partially but significantly mediated the mindfulness-creativity link for all three aspects of creative functioning, and (2) PsyCap demonstrated the strongest effect size in mediating the mindfulness-creativity link for creative problem solving, followed by creative combination and then divergent thinking. These results, on the one hand, support the positive psychology perspective by confirming a positive psychological resource mechanism regarding the relationship between mindfulness and creativity. On the other hand, the results regarding the varied sizes of the mediation effect further enrich the discourse on this perspective by showing that the mediation mechanism may function to different degrees depending on which aspect of creativity is under consideration. These findings illuminate the positive functioning of mindfulness, psychological resources/capital and creativity.

## 1 Introduction

Dispositional mindfulness and creativity have received increasing attention in both psychology research and intervention practices, as they have important implications for positive human functioning and development in a range of areas, such as health and education (Henriksen et al., 2020). While a growing body of empirical research has shown a positive link between the two constructs (He, 2023), relatively less is known about the underlying psychological mechanism by which dispositional mindfulness affects creativity (Li et al., 2023). To fill this research gap, the present study aimed to address this research question from the perspective of *positive psychology* (Seligman and Csikszentmihalyi, 2000), which postulates that dispositional mindfulness can influence creative functioning through the indirect effect (or the mediating role) of a positive psychological state (Kudesia, 2019). More specifically, this study aimed to examine the mediating role of an important component of a positive psychological state—*psychological capital* (*PsyCap*; Lorenz et al., 2022)—in directing the effect of dispositional mindfulness on creative functioning.

### 1.1 Dispositional mindfulness and creativity

Dispositional mindfulness—defined as an individual's ability to pay attention in the present moment in a nonjudgmental and nonreactive manner (Kabat-Zinn, 1994; Brown and Ryan, 2003)—has its original root in longstanding Eastern spiritual philosophy (Henriksen et al., 2020). It emerged as an important construct of positive psychology when this psychological approach was introduced at the beginning of the 21st century to direct a new trend in psychological research and interventions to emphasize the importance of positivity in human functioning (Sanchez et al., 2023). This perspective highlights that promising human functioning across multiple life domains (e.g., psychological wellbeing, achievement success, and creative functioning) can be achieved by facilitating positive human characteristics such as trait attributes and psychological strengths (Soysa et al., 2021). Under this framework, dispositional mindfulness represents one type of positive personal characteristic that can contribute to promising human functioning (Tsai et al., 2024). Indeed, research has shown that dispositional mindfulness facilitates a range of human functioning, e.g., academic achievement (Singh and Singh, 2022), cognitive gains (Kao et al., 2023), emotional regulation (O'Connor et al., 2022), psychological wellbeing (Pagnini et al., 2024), and social functioning (Deits-Lebehn et al., 2022).

In this vein, numerous studies have also documented a positive role of dispositional mindfulness in creativity (Op den Kamp et al., 2023), which is commonly conceptualized as the ability to generate ideas, insights, and/or solutions that are characterized as novel and useful (Sternberg and Lubart, 1999). For example, dispositional mindfulness has been shown to be positively associated with creative performance in a divergent thinking task, as indicated by enhanced fluency and flexibility (Berkovich-Ohana et al., 2017). Dispositional mindfulness has also been shown to be positively related to essential thinking abilities that are conducive to creativity, such as nonhabitual thinking (i.e., a greater tendency to accept new ideas; Henriksen et al., 2020), open-minded thinking (i.e., more readily accepting alternative opportunities; Deng et al., 2012), and inquisitive thinking (i.e., stronger curiosity about new inquiries; Grzybowski and Brinthaupt, 2022). In a recent study, He (2023) enriched this line of research by using the multiple-measurement approach to the multifaceted concept of creativity (Haase et al., 2018) and illustrated that dispositional mindfulness was positively predictive of three alternative aspects of creative functioning (i.e., divergent thinking, creative combination, and creative problem solving).

### 1.2 PsyCap as the mechanism that mediates the link between mindfulness and creativity

In comparison to the considerable amount of empirical research that has illustrated a positive role of dispositional mindfulness in creativity, empirical effort in uncovering the psychological mechanism that underlies this link is surprisingly scarce. The underlying psychological mechanism through which dispositional mindfulness affects creativity remains an under-researched empirical issue. Shedding light on this issue, the positive psychology perspective (Seligman and Csikszentmihalyi, 2000) postulates that positive dispositional characteristics may contribute to positive outcome functioning through their role in facilitating the development of a positive psychological state, which in turn provides sufficient psychological resources to enhance creative outcomes (Corbu et al., 2022). Put differently, the positive psychology perspective postulates a mediating role of positive psychological state (i.e., *mediator*) in linking positive dispositional characteristics (i.e., *predictor variable*) and positive human functioning (i.e., *outcome variable*).

By applying this theory to the context of creativity, researchers have taken the perspective of positive psychology and used the conservation of resource theory (Hobfoll et al., 2018) to understand the effect of dispositional mindfulness on creative functioning. According to this theory, dispositional mindfulness (a type of positive personal characteristic) can facilitate creativity (a type of positive human functioning) through the effective regulation of positive psychological resources. More specifically, dispositional mindfulness can facilitate the regulation of formulating, driving, and better managing positive psychological resources, which further contributes to positive creative outcomes (see Kudesia, 2019). According to this theory, PsyCap is emphasized as an important component of “a positive psychological state” (Luthans et al., 2007; p. 3), which provides an ideal and promising psychological context for providing sufficient positive psychological resources for boosting positive human functioning (Lorenz et al., 2022). In particular, PsyCap is highlighted as a positively oriented higher-order construct that consists of four subdimensions of positive personal characteristics (Luthans et al., 2007): (1) *self-efficacy* (i.e., confidence in one's ability to succeed at challenging tasks); (2) *optimism* (i.e., making positive attributions about success); (3) *hope* (i.e., persevering toward goals and, when necessary, redirecting paths with the hope to succeed); and (4) *resilience* (i.e., sustaining in and overcoming difficult situations to attain success; Lorenz et al., 2022). Related to the mindfulness-PsyCap-creativity relationship, the conservation of resource theory postulates that PsyCap (as a constitutive of a positive psychological state), on the one hand, is facilitated by dispositional mindfulness, while on the other hand, it provides sufficient positive psychological resources for enhancing creative functioning (Li et al., 2023). In other words, this theory represents a positive psychology perspective in postulating a mediating mechanism of PsyCap in the link between mindfulness and creativity.

Lending support to the positive psychology perspective in general and the conservation of resource theory in particular, empirical evidence has illustrated significant relationships among the three variables (i.e., dispositional mindfulness, PsyCap, and creativity) in the expected manner. For example, empirical evidence has revealed a positive role of dispositional mindfulness in enhancing PsyCap (e.g., Dirzyte et al., 2022; Gordani and Sadeghzadeh, 2023). Moreover, supporting evidence has also been found with respect to the positive role of all four subdimensions of PsyCap in enhancing creative functioning. For instance, self-efficacy, especially creative self-efficacy, has been shown to be positively linked to various aspects of creative functioning, such as divergent thinking, creative problem solving, and the use of creative cognition (e.g., Puozzo and Audrin, 2021; He, 2022). Moreover, optimism has been shown to enhance creativity by facilitating positive thinking, flexible thinking and positive affect (e.g., Lorenz et al., 2022; Lu et al., 2023). Furthermore, hope has been shown to positively impact creative performance in a variety of research contexts, including school, organizational, and clinical settings (e.g., Lorenz et al., 2022; Lei and Lei, 2023). Finally, resilience has been found to positively support creativity, especially during critical times in facing challenges (e.g., Lorenz et al., 2022; Lei and Lei, 2023; Sun et al., 2023). More importantly, PsyCap, as a higher-order construct, has been shown to better predict creative performance than each of its subcomponents (Sweetman et al., 2011; Li et al., 2023).

**Figure 1:**
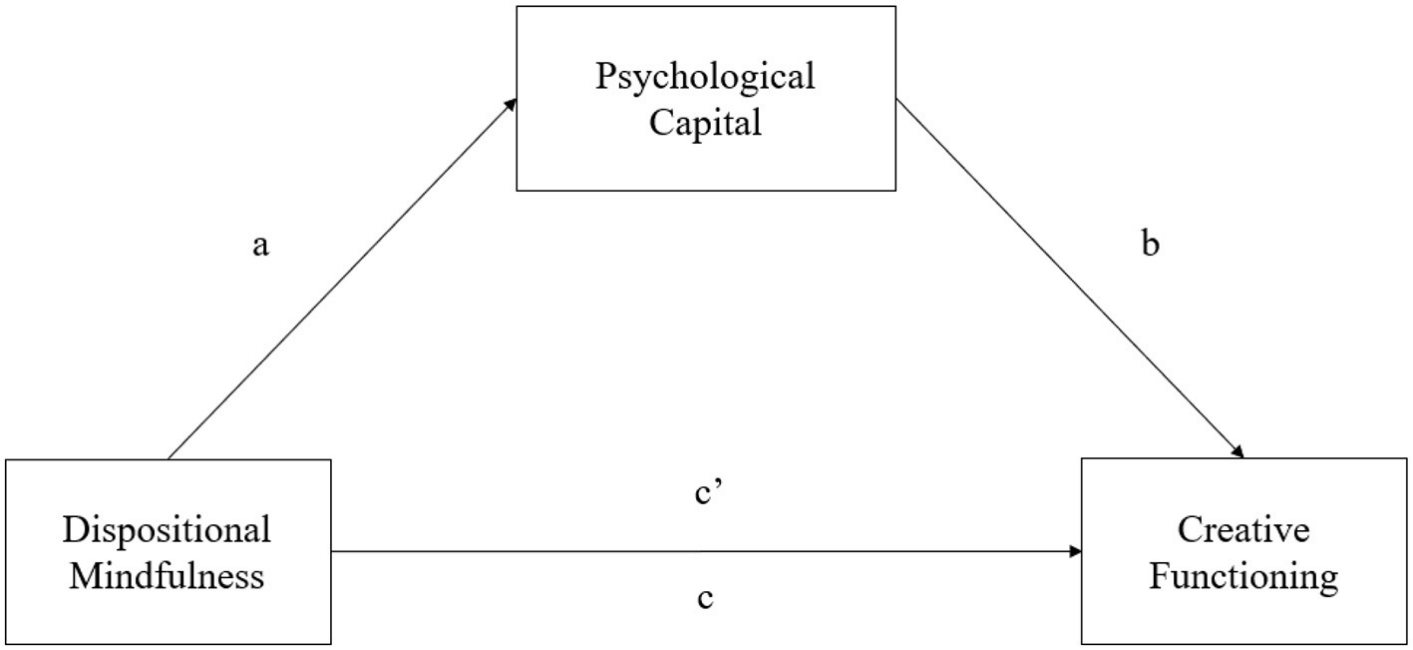
The proposed mediation relationships among the three variables, i.e., dispositional mindfulness (predictor variable), psychological capital (mediator), and creative functioning (outcome variable). *c* = total effect, *c*′ = direct effect, *a* × *b* = indirect effect.

In summary, the literature from several lines of research reviewed above presents positive links between (1) dispositional mindfulness and PsyCap, (2) PsyCap and creative functioning, and (3) dispositional mindfulness and creative functioning. The integration of these three lines of research provides empirical support for the positive psychology perspective in anticipating a mediating role of PsyCap (mediator) in directing the effect of dispositional mindfulness (predictor variable) on creative functioning (outcome variable). Figure 1 shows a diagrammatical representation of the hypothesized mediating relationships among the three variables.

### 1.3 The present study

Although an integration of several lines of research provides *indirect* empirical support for the positive psychology perspective in hypothesizing that PsyCap mediates the effect of dispositional mindfulness on creative functioning, *direct* empirical testing regarding the hypothesized mediating mechanism is surprisingly rare. Recently, Li et al. (2023) reported relevant empirical findings based on a study with an employee sample in mainland China showing that PsyCap significantly mediated the positive effect of dispositional mindfulness on employer-rated creativity through enhancing employees' creative engagement behaviors in the workplace. Notably, although Li et al. (2023) found initial empirical evidence with respect to the mediating role of PsyCap in the mindfulness-creativity link, they focused merely on one aspect of creativity in the assessment of creativity by using a six-item questionnaire to assess job supervisors' subjective ratings of their job supervisees' creative behaviors in the workplace (i.e., employer-rated creativity). However, the literature commonly acknowledges that creativity is a multifaceted construct that can be studied from multiple perspectives by using multiple creativity measures (McAleer et al., 2020).

Hence, this study aimed to extend Li et al's (2023) research by applying the multiple-measurement approach to the multifaceted concept of creativity (Haase et al., 2018) to verify the mediating role of PsyCap in the mindfulness-creativity relationship. The multiple-measurement approach to the assessment of creativity has been proven to be effective in enabling a more comprehensive evaluation of creative functioning in previous research that investigated the mindfulness-creativity relationship (He, 2023). In applying the multiple-measurement approach, the current study specifically followed Antonietti and Iannello's (2008) taxonomy regarding the assessment of creative functioning from three aspects (i.e., idea generation, combinatory ability, and restructuring ability) by using their corresponding creativity tests. First, idea generation was assessed by a divergent thinking test (i.e., the Wallach-Kogan Creativity Test/WKCT; Wallach and Kogan, 1965), which represents one of the most commonly used measures of creativity; it was designed to assess creative ability in relation to divergent production (Said-Metwaly et al., 2022). Second, combinatory ability was assessed by the Test for Creative Thinking–Drawing Production (TCT–DP, Urban and Jellen, 1996), which was developed based on a gestalt approach to creativity that aimed to tap the creative ability to combine disjointed and unrelated elements into a complete and meaningful whole in a novel and appropriate manner (He and Wong, 2011, 2022). Third, restructuring ability was assessed by a creative problem-solving test (CPST), which was designed to assess creative ability to develop a new and appropriate solution or an “aha” solution (Weisberg, 2015) to a problem as the result of a new representation and interpretation of the problem (He and Wong, 2021; He, 2023).

### 1.4 Hypotheses

Building on the perspective of positive psychology and relevant research findings, it was hypothesized that the mediating role of PsyCap in the mindfulness–creativity link, as shown in Figure 1, would be supported in three alternative aspects of creative functioning, which were assessed by (a) a divergent thinking test (i.e., the WKCT; Hypothesis 1/H1), (b) a creative combination test (i.e., the TCT–DP; Hypothesis 2/H2), and (c) a creative problem-solving test (i.e., the CPST; Hypothesis 3/H3).

## 2 Method

### 2.1 Participants and procedures

An initial sample of 916 senior secondary school students in grades 10 and 11 (51.2% female; *M*_age_ = 15.8 years, *SD* = 1.21, range = 15 to 17 years) were recruited from six coeducational secondary schools in various districts of Hong Kong, 22 of whom were excluded from the data analysis due to incomplete data (attrition rate = 2.40%). The final sample consisted of 894 participants (50.8% female; *M*_age_ = 15.5 years, *SD* = 1.14, range = 15–17 years), who had an average education level of 10.1 years (*SD* = 1.83; range = 9–11 years). All participation was voluntary. According to the information provided by the participating schools, all of these schools received government aid and admitted students from diverse backgrounds, and all of the participating students were ethnically Chinese and from middle-class or lower-middle-class socioeconomic backgrounds. Prior to data collection, all involved parties, i.e., the participating schools, the student participants and their parents, were informed about the study objectives and the confidentiality, anonymity, and safety principles of the research. Only those students who provided both informed consent from their parents and their own assent were invited to participate in the study. Informed consent was also obtained from the participating schools. During data collection, assessments of the study variables (i.e., dispositional mindfulness, psychological capital, and creative functioning) were administered to the participants using standard instructions in a group setting with ~30–40 participants in regular classrooms. The assessment procedure took ~60 min to complete.

### 2.2 Instruments

#### 2.2.1 Dispositional mindfulness

Dispositional mindfulness was assessed with the Chinese version of the Mindful Attention Awareness Scale (MAAS; Brown and Ryan, 2003; Deng et al., 2012), which consists of 15 statements that describe an individual's general tendency with respect to his or her daily awareness experiences (e.g., “I find it difficult to focus on what is happening in the present” [reverse coded]). The respondents were asked to rate their agreement on a 6-point scale (1 = almost never; 6 = almost always) to indicate the frequency of the daily awareness experiences described in the items. Higher composite scores indicate higher levels dispositional mindfulness. The MAAS is one of the most commonly used measures of trait mindfulness and has good psychometric properties, such as supporting evidence for internal consistency (e.g., α = 0.88–0.92; Mettler et al., 2019), test-retest reliability, factor structure, convergent and discriminant correlations with relevant constructs in expected directions, and change with treatment according to expected effects (see Baer, 2019; Molina-Rodríguez et al., 2023). Evidence supporting the good psychometric properties of the Chinese MAAS has been reported in several studies involving Chinese student samples (Deng et al., 2012; He, 2023; Li et al., 2023). A high internal consistency of the scale (α = 0.90) was also found in this study.

#### 2.2.2 PsyCap

To assess PsyCap, the revised Compound Psychological Capital Scale (CPC-12R; Dudasova et al., 2021; Lorenz et al., 2022) was adapted and translated into Chinese using a standard back-translation procedure. The scale was first translated into Chinese by a bilingual and experienced researcher in the field of educational psychology. The Chinese version of the scale was subsequently back-translated into English by a bilingual postdoctoral fellow in educational psychology. The back-translated version was then compared to its original version to evaluate the consistency between the two versions, and revisions were repeated until satisfactory consistency was achieved. The Chinese CPC-12R consists of 12 items, with three items assessing each of the four subscales (i.e., self-efficacy, hope, optimism, and resilience), which constitute the higher-order factor “PsyCap.” Sample items include “I can solve most problems if I invest the necessary effort” (self-efficacy), “If I should find myself in a jam, I could think of many ways to get out of it” (hope), “Overall, I expect more good than bad things to happen to me” (optimism), and “I consider myself to be able to stand a lot, I am not easily discouraged by failure” (resilience). Participants responded on a 6-point scale with answers ranging from 1 (strongly disagree) to 6 (strongly agree) to indicate the extent to which they agreed with the item statements. PsyCap was calculated by taking the average of the total scores of the four subscales. Higher scores indicate greater PsyCap.

The scale was first validated in Czech and Slovak samples, for which good psychometric properties of the scale were reported (Dudasova et al., 2021; Lorenz et al., 2022). The good psychometric properties of the scale with respect to its construct validity, concurrent validity, convergent validity and discriminant validity were subsequently supported in a large-scale study involving American, Czech, and Slovak samples (Prochazka et al., 2023) and in a Japanese sample (Ikeda et al., 2023). Because this is the first study that has applied the scale in a Chinese student sample, the construct validity of the CPC-12R was analyzed using a confirmatory factor analysis (CFA) with four first-order factors (i.e., self-efficacy, hope, optimism, and resilience), and one higher-order factor (i.e., PsyCap) was identified. The obtained fit indices of the resulting model (*CFI* = 0.942, *TLI* = 0.929, *RMSEA* = 0.049, *SRMR* = 0.057) supported the construct validity of the scale in the current Chinese sample, although the chi-square value was statistically significant (χ^2^ = 204.81, *df* = 50, *p* < 0.001). Moreover, good internal consistency with α = 0.88 was also found for the PsyCap scale in the current sample.

#### 2.2.3 Creativity

##### 2.2.3.1 Divergent thinking test

The Chinese version of the WKCT (Wallach and Kogan, 1965) was applied to assess the ability to generate diverse and numerous ideas. The WKCT has well-supported psychometric properties in Chinese student samples (Cheung et al., 2004; Cheung and Lau, 2013; He and Wong, 2021). The Chinese WKCT applied in the present study consists of both verbal and figural test items. The verbal test items consist of (1) alternate uses (i.e., name as many ways as possible that you could use a newspaper) and (2) instances (i.e., name as many things as possible that have wheels). The figural test items consist of (1) line meanings (i.e., name as many things or meanings as possible that the given line makes you think of) and (2) pattern meanings (i.e., name as many things or meanings as possible that the given pattern makes you think of). Participants were given 5 min to respond to each item. Idea generation was evaluated by two indices of divergent thinking: (1) fluency (i.e., the total number of nonredundant responses) and (2) flexibility (i.e., the number of categories in which the given responses could be categorized). All the responses were coded by two experienced creativity researchers, and the average scores of the two coders were used for the data analyses. The intraclass correlation coefficients (ICCs) suggested good interrater reliability, with all the ICCs >0.90 (ICC_Verbal_fluency_ = 0.95; ICC_Verbal_flexiblity_ = 0.93; ICC_Figural_fluency_ = 0.96; ICC_Figural_flexiblity_ = 0.92; all *p-values* < 0.001). Moreover, good internal consistency of the test was also found in this study, with α = 0.81–0.84 (see Table 1).

**Table 1:** Means, standard deviations, Cronbach's alpha coefficients, and correlation coefficients of the study variables.

	***M***	***SD***	**α**	**1**	**2**	**3**	**4**	**5**	**6**	**7**	**8**	**9**	**10**	**11**	**12**
1. Age	15.6	1.14	–	1											
2. Gender	0.49	0.50	–	0.02	1										
3. Education	10.1	1.83	–	0.91^***^	0.03	1									
4. Dispositional mindfulness	3.12	1.28	0.90^**^	0.04	0.12^*^	0.06	1								
5. PsyCap	3.16	1.41	0.88^**^	0.03	0.10	0.08	0.43^***^	1							
6. WKCT-Fluency (verbal)	19.3	9.88	0.84^**^	0.09	0.14^*^	0.13^*^	0.19^**^	0.27^**^	1						
7. WKCT-Flexibility (verbal)	3.48	1.67	0.82^**^	0.12^*^	0.13^*^	0.12^*^	0.21^**^	0.25^**^	0.41^***^	1					
8. WKCT-Fluency (figural)	18.4	10.6	0.83^**^	0.10	0.11	0.15^*^	0.20^**^	0.26^**^	0.28^**^	0.16^*^	1				
9. WKCT-Flexibility (figural)	3.11	1.73	0.81^**^	0.13^*^	0.10	0.13^*^	0.22^**^	0.23^**^	0.23^**^	0.18^**^	0.40^***^	1			
10. TCT–DP	19.8	10.1	0.89^**^	0.08	0.15^*^	0.12^*^	0.31^**^	0.34^**^	0.13^*^	0.14^*^	0.14^*^	0.15^*^	1		
11. CPST (verbal)	58.1	10.2	0.82^**^	0.13^*^	0.14^*^	0.15^*^	0.41^***^	0.46^***^	0.10	0.09	0.06	0.09	0.13^*^	1	
12. CPST (figural)	54.0	9.79	0.81^**^	0.14^*^	0.10	0.14^*^	0.40^***^	0.47^***^	0.08	0.07	0.08	0.10	0.15^*^	0.23^**^	1

Gender is dummy coded: 0 = male; 1 = female.

WKCT, Wallach-Kogan Creativity Test; TCT–DP, Test for Creative Thinking–Drawing Production; CPST, Creative Problem Solving Test.

^***^p <0.001.

^**^p <0.01.

^*^p <0.05.

##### 2.2.3.2 The Test for Creative Thinking–Drawing Production

The Chinese adapted TCT–DP (Form A, Urban and Jellen, 1996) was applied to assess the ability to combine fragmental components into a holistic meaningful whole in an original and appropriate manner (He and Wong, 2011). In particular, the test assesses creative thinking through a drawing task on an A4-sized testing sheet that contains six intriguing figural fragments: (a) a semicircle, (b) a point, (c) a 90° angle, (d) a curved line, (e) a broken line, and (f) a small open square. The drawing can be completed using any combination of the six figural fragments in a wide variety of ways, ranging from simple, conventional, and disjointed combinations to thematically complex, unconventional, integrated, and aesthetically interesting combinations. The test has been highlighted as a promising instrument for assessing creative thinking based on a componential and holistic approach (He, 2023). Evidence for the psychometric properties of the test has been well documented in Chinese student samples (He and Wong, 2015, 2022).

Based on the TCT–DP test manual, creative thinking was scored according to nine criteria [i.e., continuation, completion, new elements, connections by line, connections by theme, boundary breaking (consisting of two subcriteria), perspective, humor and affectivity, and unconventionality (consisting of four subcriteria)], with 0–66 points as the total possible score range. Higher composite scores indicate greater creative functioning. A rigorous rater training process was carried out with two experienced creativity researchers by using example drawings from other datasets to ensure accurate and reliable scoring. The two trained raters then scored all the TCT–DP protocols, and their average scores were used for data analyses. High interrater reliability (ICC = 0.94) and internal consistency (α = 0.89) were obtained for the TCT–DP composite score.

##### 2.2.3.3 Creative problem-solving test

The 10-item CPST (five verbal and five figural problems; Lin et al., 2012) was employed in this study to assess the restructuring ability for the sudden realization of a new and appropriate approach to a problem (Weisberg, 2015). Support for the psychometric properties of the CPST in Chinese student samples has been well-documented (He and Wong, 2021; He, 2023; Lin, 2023). A sample item of the verbal problems is “Erin stumbles across an abandoned cabin one cold, dark and snowy night. Inside the cabin are a kerosene lantern, a candle, and wood in a fireplace. She has only one match. What should she light first?” A sample item of the figural problems is “Nine pigs are kept in a square pen. Build two more square enclosures that would put each pig in a pen by itself” (see Lin et al., 2012, p. 122–123, for complete test items). Participants were given 20 min to complete this task. The performance scores for creative problem solving were calculated as the percentage of problems that were answered correctly within the verbal and figural items. Good internal consistency of the scale was obtained for both verbal (α = 0.81) and figural (α = 0.82) items in this study.

### 2.3 Data analysis

Prior to hypothesis testing, descriptive analyses of all study variables were performed to determine data normality and sample characteristics by using SPSS 28.0, with the statistical significance level set at *p* < 0.05. Normality tests were conducted to confirm that all study variables were within the range of normal distribution (skewness = −0.41–0.48 and kurtosis = 0.50–0.68; Shanthi, 2019). Moreover, a Pearson product-moment correlation analysis was performed to determine whether the anticipated bivariate correlation could be found among the study variables (i.e., dispositional mindfulness, PsyCap, and creative functioning). In hypothesis testing, a mediation approach of the bootstrapping method with 5,000 samples (Preacher and Hayes, 2008) using Amos 28.0 was applied to examine whether the effect of dispositional mindfulness on each outcome variable regarding creative functioning was mediated by PsyCap, in which context participants' demographic variables (i.e., age, education, and gender) were included in the analysis to control for their potential confounding effect. The indirect mediation effect on each outcome variable indicated statistical significance when the 95% confidence intervals (CIs) did not include zero (Abu-Bader and Jones, 2021).

## 3 Results

### 3.1 Bivariate correlations

The descriptive statistics and the correlation matrix of the study variables are presented in Table 1. Related to the hypotheses regarding the mediating role of PsyCap in the links between dispositional mindfulness and three aspects of creative functioning, the results of the correlation coefficients revealed that dispositional mindfulness was positively correlated with PsyCap (*r* = 0.43) and all of the creativity scores in the three creativity tests, including the WKCT (*r* = 0.19–0.22), the TCT–DP (*r* = 0.31), and the CPST (*r* = 0.40–0.41), at a statistically significant level of *p* < 0.01. Furthermore, significant results were also found with respect to the positive bivariate correlations between PsyCap and all of the creativity scores in the WKCT (*r* = 23–0.27), the TCT–DP (*r* = 0.34), and the CPST (*r* = 0.46–0.47), with all *p*-values <0.01. These results support the anticipated bivariate correlations among the three study variables (i.e., dispositional mindfulness, PsyCap, and creative functioning). Moreover, the bivariate correlations among the three creativity measures are weak or approaching zero (*r* = 0.06–0.15), supporting the claim that the three creativity measures focus on different aspects of creative functioning.

### 3.2 Mediation analyses

**Figure 2:**
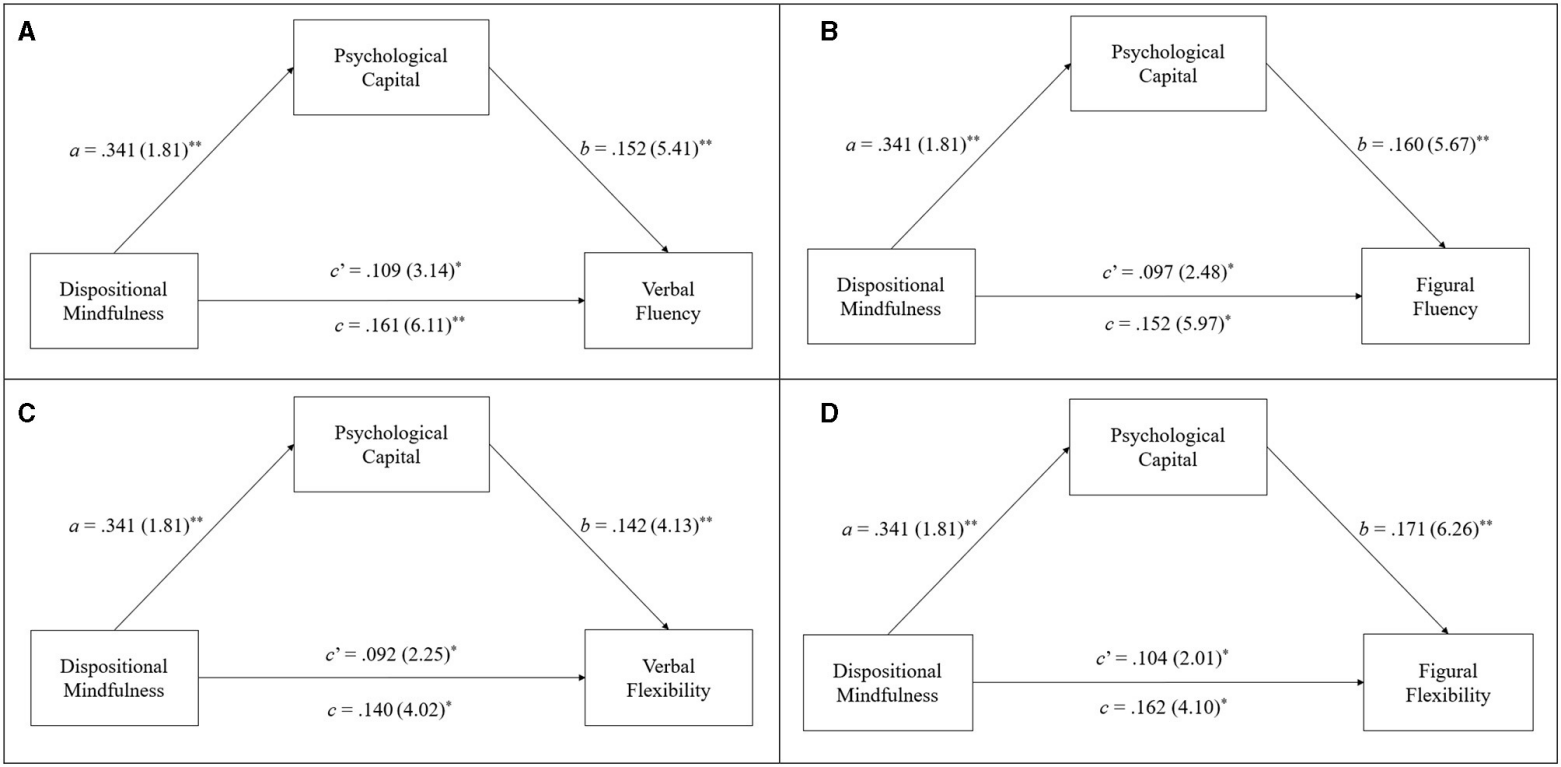
Results of mediation analyses on WKCT scores regarding verbal fluency **(A)**, figural fluency **(B)**, verbal flexibility **(C)**, and figural flexibility **(D)**. Standardized path coefficient are shown, with corresponding unstandardized coefficients in parentheses. **p < 0.01, *p < 0.05.

**Figure 3:**
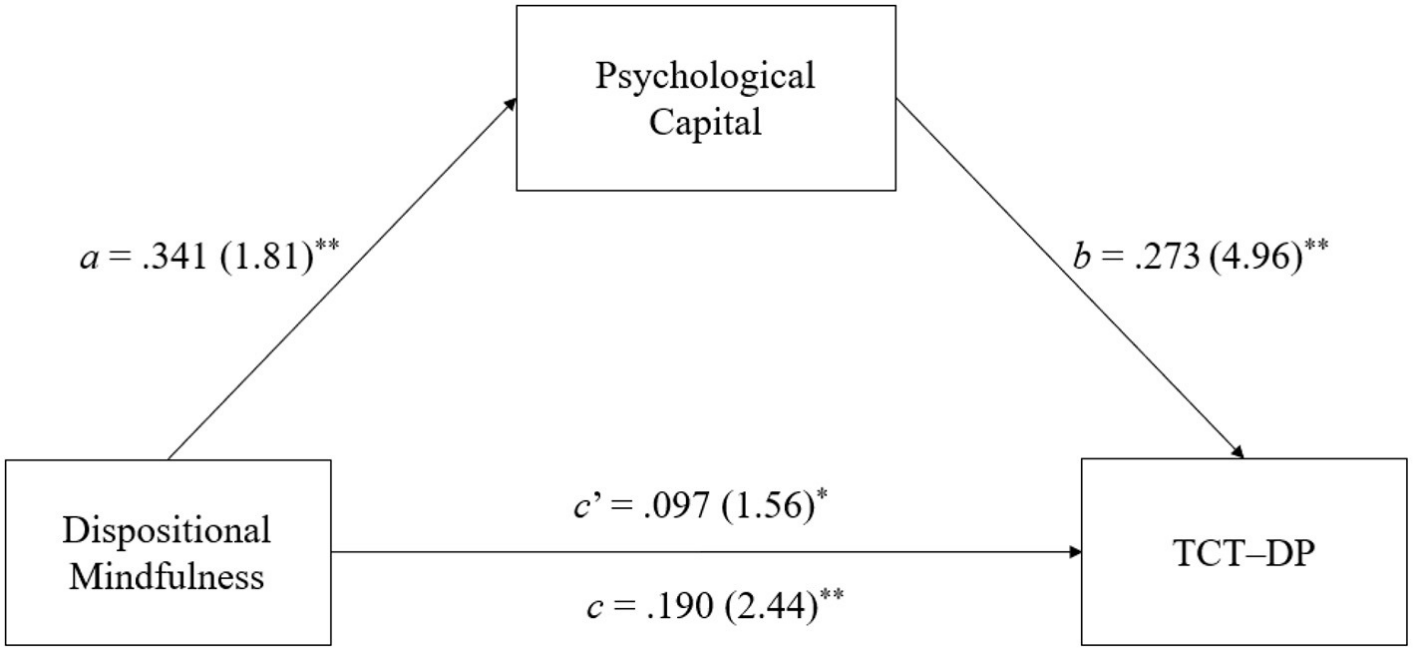
Results of mediation analyses on the TCT-DP score. Standardized path coefficients are shown, with corresponding unstandardized coefficients in parentheses. **p < 0.01, *p < 0.05.

**Figure 4:**
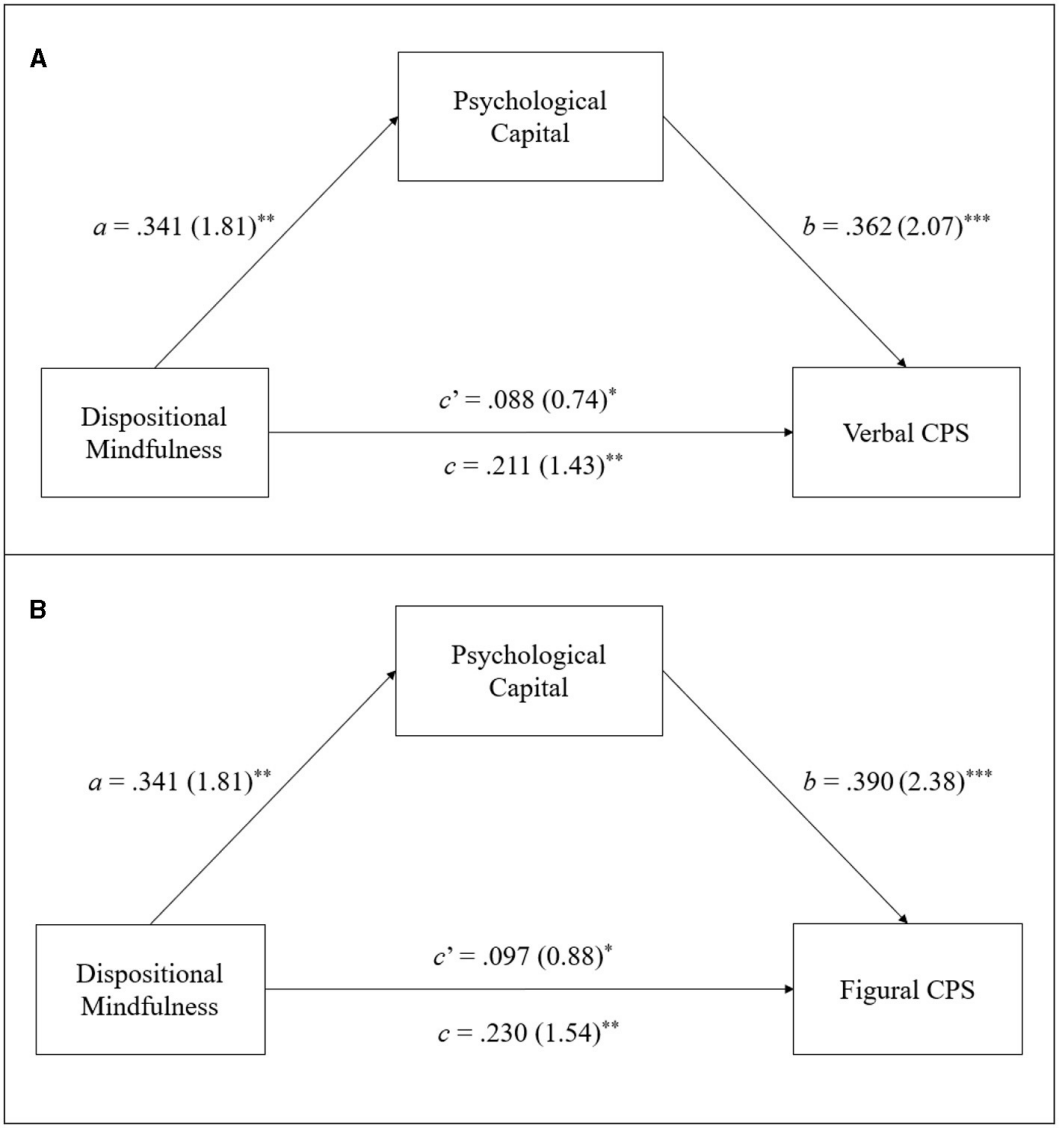
Results of mediation analyses on the CPST scores regarding verbal CPS **(A)** and figural CPS **(B)**. Standardized path coefficient are shown, with corresponding unstandardized coefficients in parentheses. ***p < 0.001, **p < 0.01, *p < 0.05.

With respect to the hypothesis testing regarding the mediating role of PsyCap in the mindfulness-creativity relationship in terms of the three aspects of creative functioning, i.e., divergent thinking, creative combination, and creative problem solving, the results of the bootstrapped estimates of the total (*c*) and direct (*c*′) effects of dispositional mindfulness on creative functioning and its indirect (*a* × *b*) effect on creative functioning via PsyCap are presented in Tables 2–4, respectively. Moreover, Figures 2–4 provide a visual representation of the relevant results. Below is a summary of the results according to the three hypotheses.

**Table 2:** Results of mediation analyses on the WKCT scores.

**Path/effect**	**Bootstrap estimate**		**95% CI**	**P_M_**
	β	***SE***	**Bootstrap with bias correction**	
*c* (Dispositional mindfulness → verbal fluency)	0.161^**^	0.114	(0.094, 0.219)	32.3%
*a* (Dispositional mindfulness → PsyCap)	0.341^**^	0.129	(0.113, 0.552)	
*b* (PsyCap → verbal fluency)	0.152^**^	0.108	(0.094, 0.219)	
*c*′	0.109^*^	0.097	(0.077, 0.116)	
*a × b*	0.052^*^	0.025	(0.009, 0.041)	
*c* (Dispositional mindfulness → figural fluency)	0.152^**^	0.103	(0.088, 0.221)	36.2%
*a* (Dispositional mindfulness → PsyCap)	0.341^**^	0.129	(0.113, 0.552)	
*b* (PsyCap → verbal flexibility)	0.160^**^	0.110	(0.090, 0.224)	
*c*′	0.097^*^	0.066	(0.064, 0.119)	
*a × b*	0.055^*^	0.021	(0.001, 0.098)	
*c* (Dispositional mindfulness → verbal flexibility)	0.140^**^	0.100	(0.074, 0.211)	34.3%
*a* (Dispositional mindfulness → PsyCap)	0.341^**^	0.129	(0.113, 0.552)	
*b* (PsyCap → figural fluency)	0.142^**^	0.101	(0.069, 0.230)	
*c*′	0.092^*^	0.071	(0.057, 0.153)	
*a × b*	0.048^*^	0.020	(0.002, 0.091)	
*c* (Dispositional mindfulness → figural flexibility)	0.162^**^	0.099	(0.098, 0.253)	35.8%
*a* (Dispositional mindfulness → PsyCap)	0.341^**^	0.129	(0.113, 0.552)	
*b* (PsyCap → figural flexibility)	0.171^**^	0.119	(0.061, 0.224)	
*c*′	0.104^*^	0.089	(0.017, 0.199)	
*a × b*	0.058^*^	0.029	(0.001, 0.148)	

WKCT, Wallach-Kogan Creativity Test; PsyCap, Psychological Capital; c, total effect; c′, direct effect; a × b, indirect effect; 95% CI, 95% confidence interval; P_M_, proportion of the total effect for which the mediator accounts.

^**^p <0.01.

^*^p <0.05.

**Table 3:** Results of mediation analyses on the TCT–DP score.

**Path/effect**	**Bootstrap estimate**		**95% CI**	**P_M_**
	β	***SE***	**Bootstrap with bias correction**	
*c* (Dispositional mindfulness → TCT–DP)	0.190^**^	0.103	(0.072, 0.277)	48.9%
*a* (Dispositional mindfulness → PsyCap)	0.341^**^	0.129	(0.094, 0.219)	
*b* (PsyCap → TCT–DP)	0.273^**^	0.117	(0.118, 0.403)	
*c*′	0.097^*^	0.051	(0.021, 0.196)	
*a × b*	0.093^*^	0.062	(0.017, 0.208)	

TCT–DP, test for creative thinking–drawing production; PsyCap, psychological capital; c, total effect; c′, direct effect; a × b, indirect effect; 95% CI, 95% confidence interval; P_M_, proportion of the total effect for which the mediator accounts.

^**^p <0.01.

^*^p <0.05.

**Table 4:** Results of mediation analyses on the CPST scores.

**Path/effect**	**Bootstrap estimate**		**95% CI**	**P_M_**
	β	***SE***	**Bootstrap with bias correction**	
*c* (Dispositional mindfulness → verbal CPS)	0.211^**^	0.114	(0.107, 0.399)	58.3%
*a* (Dispositional mindfulness → PsyCap)	0.341^**^	0.129	(0.094, 0.219)	
*b* (PsyCap → verbal CPS)	0.362^**^	0.108	(0.184, 0.563)	
*c*′	0.088^*^	0.097	(0.031, 0.207)	
*a* × *b*	0.123^*^	0.025	(0.080, 0.211)	
*c* (Dispositional mindfulness → figural CPS)	0.230^**^	0.100	(0.141, 0.370)	57.8%
*a* (Dispositional mindfulness → PsyCap)	0.341^**^	0.129	(0.094, 0.219)	
*b* (PsyCap → figural CPS)	0.390^**^	0.121	(0.112, 0.503)	
*c*′	0.097^*^	0.066	(0.031, 0.186)	
*a* × *b*	0.133^*^	0.077	(0.057, 0.227)	

CPST, Creative Problem Solving Test; PsyCap, Psychological Capital; c, total effect; c′, direct effect; a × b, indirect effect; 95% CI, 95% Confidence Interval; P_M_, proportion of the total effect for which the mediator accounts.

^**^p <0.01.

^*^p <0.05.

#### 3.2.1 Hypothesis 1

H1 posits that PsyCap mediates the effect of dispositional mindfulness on divergent thinking as measured by the WKCT. The results of the path coefficients (i.e., the β statistics) shown in Table 2 and Figure 2 illustrate that dispositional mindfulness (i.e., predictor variable) has a significant total effect on all four outcome variables of divergent thinking (i.e., the WKCT scores), including (1) verbal fluency (*c* = 0.161, *SE* = 0.114), (2) figural fluency (*c* = 0.152, *SE* = 0.103), (3) verbal flexibility (*c* = 0.140, *SE* = 0.100), and (4) figural flexibility (*c* = 0.162, *SE* = 0.099). All *p*-values <0.01. Moreover, the results further reveal a significant indirect effect of PsyCap through which dispositional mindfulness affects all of the outcome variables of divergent thinking. In particular, dispositional mindfulness was found to positively predict PsyCap (*a* = 0.341, *SE* = 0.129), while PsyCap positively predicts all four WKCT scores, including verbal fluency (*b* = 0.152, *SE* = 0.108), figural fluency (*b* = 0.160, *SE* = 0.110), verbal flexibility (*b* = 0.142, *SE* = 0.101), and figural flexibility (*b* = 0.171, *SE* = 0.119). All *p*-values <0.01. Supporting H1, the results of the indirect effect (*a* × *b*) found by reference to 5,000 bootstrap samples revealed that the 95% CI did not include zero, which suggests that PsyCap significantly mediates the influence of dispositional mindfulness on all four WKCT scores, including verbal fluency [*a* × *b* = 0.052, *SE* = *0.0*25, 95% CI = (0.009, 0.041)], figural fluency [*a* × *b* = 0.055, *SE* = *0.0*21, 95% CI = (0.001, 0.098)], verbal flexibility [*a* × *b* = 0.048, *SE* = *0.0*20, 95% CI = (0.002, 0.091)], and figural flexibility [*a* × *b* = 0.058, *SE* = 0.029, 95% CI = (0.001, 0.148)].

Moreover, the results with respect to the direct effect of dispositional mindfulness on all four WKCT scores (i.e., the *c*′ statistics) remained statistically significant after controlling for the indirect effect of PsyCap (for verbal fluency; *c*′ = 0.109, *SE* = 0.097; for figural fluency; *c*′ = 0.097, *SE* = 0.066; for verbal flexibility: *c*′ = 0.092, *SE* = 0.071; for figural flexibility: *c*′ = 0.058, *SE* = 0.029). All *p*-values <0.05. These results suggest that PsyCap only partially mediates the influence of dispositional mindfulness on divergent thinking, although this effect was statistically significant. Further estimations of the mediation effect revealed that PsyCap was able to direct ~32.3% of the total effect of dispositional mindfulness on verbal fluency [P_M_ = (0.052)/(0.161) × 100%], 36.2% of the effect on figural fluency [P_M_ = (0.055)/(0.152) × 100%], 34.3% of the effect on verbal flexibility [P_M_ = (0.048)/(0.140) × 100%], and 35.8% of the effect on figural flexibility [P_M_ = (0.058)/(0.162) × 100%].

#### 3.2.2 Hypothesis 2

H2 posits PsyCap to mediate the effect of dispositional mindfulness on the TCT–DP score, which measures creative combination. The path coefficients shown in Table 3 and Figure 3 reveal that dispositional mindfulness significantly and positively affects both the DV (TCT–DP score; *c* = 0.190, *SE* = 0.103, *p* < 0.01) and the mediator (PsyCap; *a* = 0.341, *SE* = 0.129, *p* < 0.01). In addition, the mediator PsyCap significantly and positively impacts the TCT–DP score (*b* = 0.273, *SE* = 0.117, *p* < 0.01). In support of H2, the results regarding the indirect effect (*a* × *b* = 0.093, *SE* = 0.062) found by reference to 5,000 bootstrap samples indicate a significant indirect mindfulness-creativity relationship mediated by PsyCap because the 95% CI [0.017, 0.208] did not include zero. Furthermore, the *c*′ statistics showed that the direct effect of dispositional mindfulness on the TCT–DP score (*c*′ = 0.097, *SE* = *0.0*51, *p* < 0.05) remained statistically significant after controlling for the indirect effect of PsyCap, suggesting that PsyCap only partially mediates the impact of dispositional mindfulness on creative combination. More specifically, PsyCap was found to mediate ~48.9% of the total effect of dispositional mindfulness on creative combination [P_M_ = (0.093)/(0.190) × 100%].

#### 3.2.3 Hypothesis 3

Regarding H3, which posits PsyCap to mediate the effect of dispositional mindfulness on creative problem solving, the path coefficients shown in Table 4 and Figure 4 reveal that dispositional mindfulness (IV) significantly and positively affects both DVs: verbal (*c* = 0.211, *SE* = 0.114, *p* < 0.01) and figural CPST scores (*c* = 0.230, *SE* = *0.1*00, *p* < 0.01). Moreover, dispositional mindfulness also significantly and positively affects the mediator (PsyCap; *a* = 0.341, *SE* = 0.129, *p* < 0.01). In addition, the mediator PsyCap significantly and positively impacts both verbal (*b* = 0.362, *SE* = 0.108, *p* < 0.01) and figural CPST scores (*b* = 0.390, *SE* = 0.121, *p* < 0.01). Consistent with H3, the results regarding the indirect effect found by reference to 5,000 bootstrap samples indicate that PsyCap significantly mediates the mindfulness-creativity relationship for both verbal [*a* × *b* = 0.123, *SE* = 0.025, 95% CI = (0.080, 0.211)] and figural creative problem solving [*a* × *b* = 0.133, *SE* = *0.0*77, 95% CI = (0.057, 0.227)] because the 95% CI did not include zero. Moreover, because the direct effect of dispositional mindfulness on both verbal (*c*′ = 0.088, *SE* = 0.097, *p* < 0.05) and figural creative problem solving (*c*′ = 0.097, *SE* = 0.066, *p* < 0.05) remained significant after controlling for the indirect effect of PsyCap, the results suggest that PsyCap only partially mediates the influence of dispositional mindfulness on both verbal and figural creative problem solving. In particular, PsyCap mediated ~58.3% and 57.8% of the total effect of dispositional mindfulness on verbal [P_M_ = (0.123)/(0.211) × 100%] and figural creative problem solving [P_M_ = (0.133)/(0.230) × 100%], respectively.

## 4 Discussion

From the perspective of positive psychology (Seligman and Csikszentmihalyi, 2000), the present study examined the mediating role of PsyCap with respect to the effect of dispositional mindfulness on creative functioning. By adopting the multiple-measurement approach to creativity (Haase et al., 2018; He, 2023), the hypothesized mediating role of PsyCap in the mindfulness–creativity link was examined in three aspects of creative functioning (i.e., divergent thinking, creative combination, and creative problem solving), which were measured by the WKCT, the TCT–DP, and the CPST, respectively. The major findings of the study are highlighted and discussed below.

### 4.1 PsyCap partially and significantly mediates the mindfulness-creativity link for three aspects of creative functioning

The results of the present study revealed a general pattern in which higher levels of dispositional mindfulness were associated with higher levels of PsyCap, which in turn contributed to enhanced performance in three aspects of creative functioning (i.e., divergent thinking, creative combination, and creative problem solving). The results of mediation analyses using a bootstrapping approach based on 5,000 samples (Preacher and Hayes, 2008) further confirmed a partial but significant mediating role of PsyCap in the influences of dispositional mindfulness on (1) divergent thinking (i.e., supporting H1), (2) creative combination (i.e., supporting H2), and (3) creative problem solving (i.e., supporting H3). These results, on the one hand, align well with many previous findings of positive links between (a) dispositional mindfulness and creative functioning (e.g., Henriksen et al., 2020; Grzybowski and Brinthaupt, 2022; He, 2023), (b) dispositional mindfulness and PsyCap (e.g., Dirzyte et al., 2022; Gordani and Sadeghzadeh, 2023), and (c) PsyCap and creative functioning (e.g., He and Wong, 2022; Lorenz et al., 2022; Lei and Lei, 2023; Sun et al., 2023).

On the other hand, the finding that PsyCap significantly mediates the effect of dispositional mindfulness on creative functioning are consistent with those reported by Li et al. (2023), who presented initial empirical evidence to support PsyCap as the mediating mechanism underlying the link between mindfulness and creativity. More importantly, while Li et al. (2023) study of an adult sample of employees in mainland China used a scale-based measurement that captured employers' subjective ratings regarding their subordinates' creativity in the workplace, the present study extends Li et al's (2023) work by generalizing their findings to (a) a younger age group of Chinese students in Hong Kong and (b) three aspects of creative functioning using three types of objective performance-based creativity tests in school settings (i.e., the WKCT, the TCT–DP, and the CPST). Altogether, the findings based on different samples (i.e., adults or adolescents, employees or students) and different creativity measures (i.e., subjective ratings or objective performance) support the positive psychology perspective by showing that one reason dispositional mindfulness facilitates creative functioning is that the positive state of consciousness or psychological context resulting from trait mindfulness is likely to increase one's psychological capital to provide sufficient psychological resources to boost creative performance.

These findings illuminate the understanding of the mechanism underlying the effect of dispositional mindfulness on creativity. Many past studies have attempted to understand the role of mindfulness in creativity from a cognitive processing perspective, which claims that mindfulness is conducive to creativity because it is associated with enhanced cognitive processing abilities such as switching thinking perspectives (e.g., Carson and Langer, 2006), responding in a nonhabitual manner (Moore and Malinowski, 2009), increasing capacity in working memory (Chiesa et al., 2011), facilitating concentration during task engagement (Khan and Abbas, 2022), and reducing fearful thoughts about being judged (Carson and Langer, 2006). The findings of the present study, together with those reported by Li et al. (2023), add new knowledge to the mindfulness-creativity literature by presenting new empirical findings supporting an alternative explanation from the perspective of positive psychology, which argues for a mechanism of psychological capital/resources that buttresses the mindfulness-creativity relationship. These findings enrich the scientific understanding by identifying plausible psychological mechanisms underlying the effect of positive trait characteristics on positive functioning outcomes.

### 4.2 PsyCap has varied mediating power for the various aspects of creative functioning

Another new and important finding of the present study was related to the varied size of the mediation effect of PsyCap on the link between mindfulness and creativity across the various aspects of creative functioning. By adopting the multiple-measurement approach to creativity and investigating the mediating effect of PsyCap on the mindfulness-creativity link in a single study, evidence enriching the positive psychology perspective was found by showing that PsyCap does not mediate the effect of dispositional mindfulness on the various aspects of creative functioning to the same degree. Rather, it shows different mediation powers regarding the mindfulness-creativity link depending on the aspect of creativity considered. Specifically, the findings generated in this study suggest that PsyCap has the strongest mediating effect on the impact of dispositional mindfulness on creative problem solving, followed by creative combination, and the weakest mediating effect on divergent thinking. In this student sample, PsyCap was found to mediate 57.8%−58.7% of the total effect of dispositional mindfulness on creative problem solving, 48.9% of the effect on creative combination, and 32.3%−36.2% of the effect on divergent thinking. These results are interesting when He (2023) research findings are considered, in which the influence of dispositional mindfulness was also found to vary across aspects of creative functioning, with its explanatory power for creative problem solving (*R*^2^ = 11–13%) being greater than that for creative combination (*R*^2^ = 9%) and then divergent thinking (*R*^2^ = 4%−6%). Taken together, the findings of this current study and those reported by He (2023) converge to suggest an interesting pattern in which either dispositional mindfulness or psychological capital has a stronger impact on creative problem solving than on creative combination and subsequently on divergent thinking.

However, although it is an interestingly new finding with respect to the varied mediating effect sizes of PsyCap on the mindfulness-creativity link, the data obtained in the present study have limitations in explaining why the mediating effect varies across aspects of creative functioning. One reason may be related to the multidimensional nature of creativity and PsyCap, in which the various aspects of creative functioning captured by different types of creativity measurement (e.g., divergent thinking test, creative combination test, creative problem test) may rely on different kinds of psychological resources (e.g., self-efficacy, optimism, hope, and resilience) to different extents. Moreover, trait mindfulness and psychological resources may also contribute differently to different creative processes or components, with a stronger effect on certain processes or components than on others. For example, past mindfulness research has shown that trait mindfulness may have different effects on different creative stages or processes (Henriksen et al., 2020). In particular, Lebuda et al. (2016) reported that the effect of trait mindfulness was greater for creativity measurements involving insight problem-solving tasks than for those involving divergent thinking tasks. In addition, research has illustrated that PsyCap, as a higher-order positive psychological construct that consists of four subdimensions (i.e., self-efficacy, optimism, hope, and resilience), is complex in that it promotes creativity (Yu et al., 2019; Yan et al., 2020), with some of its subcomponents contributing more to a certain aspect of creativity than others (e.g., Puozzo and Audrin, 2021; He and Wong, 2022; Lorenz et al., 2022; Lei and Lei, 2023; Sun et al., 2023). While past studies tend to support the varied mediating power of PsyCap on the link between mindfulness and creativity in relation to the multidimensional nature of creativity and PsyCap, future research is warranted to verify this speculative explanation.

### 4.3 Limitations and directions for future research

Although the current study generates interesting and important new findings to illuminate the psychological mechanism underlying the effect of dispositional mindfulness on creativity, some limitations should be noted in the interpretation of the results. The first limitation concerns the cross-sectional design, which is generally considered not as robust as an experimental design. As such, the findings and their interpretation with respect to the mediating role of PsyCap in the mindfulness-creativity link might be less than conclusive, given the common limitation of a cross-sectional design (Sande and Ghosh, 2018). However, when dealing with the variable in relation to stable personal traits (e.g., dispositional mindfulness), a study design using experimental manipulation appears to make less sense. However, experimental paradigms may be used in a future study by inducing higher levels of state mindfulness through mindfulness interventions (e.g., mediation, priming) to examine whether PsyCap still mediates the effect of the induced higher levels of state mindfulness on creative functioning. Second, as all participants involved in this study were Chinese adolescents studying in secondary schools in Hong Kong who have a limited age range of 15–17 years, it is interesting to further examine whether these findings can be generalized to other age groups (e.g., adults, elderly, and young children). It is also warranted to further examine whether these findings can be generalized to other populations with different ethnic and socioeconomic backgrounds. Hence, future research should recruit other types of participants with different demographic, educational, ethnic, and socioeconomic backgrounds, to test the generalizability of the findings. Third, while a multiple-measurement approach was applied to assess creativity with three types of creativity tests, a single measurement was used to assess PsyCap (i.e., the CPC-12R) and dispositional mindfulness (the MAAS). However, these two constructs were also considered to have multidimensional characteristics (e.g., He, 2023; Wrahatnolo and Anistyasari, 2023). Future research should use various types of PsyCap and dispositional mindfulness measures with the aim of testing the generalizability of the research findings obtained by the current study.

Fourth, while this study focused on mediation mechanism of psychological capital that underlies the link between dispositional mindfulness and creative functioning, it is interesting to further explore whether or not psychological capital also contributes to the psychological mechanism that underlies the effect of mindfulness interventions on creative outcomes. Although more and more researchers and educational practitioners have highlighted the impact of mindfulness interventions (e.g., short-term and long-term mindfulness-training and mindfulness-practice) on creative performance in school settings (Tastanova et al., 2024), the underlying psychological mechanism through which mindfulness interventions could benefit creative outcomes remains an under-researched empirical question. Future empirical studies may extend the research findings of the present study to examine whether psychological capital serves as the underlying mediator in explaining the beneficial effect of mindfulness interventions on creative outcomes. Finally, while the present study applied the positive psychology perspective to the context of creativity and found empirical evidence to support the mediating role of psychological capital in the mindfulness-creativity link, the positive psychology perspective has a wider spectrum in expecting a mediating role of positive psychological state in linking more positive dispositional characteristics and more positive human functioning. Future studies should extend the research with respect to the mediation mechanism of psychological capital to other types of positive dispositional characteristics and positive human functioning and verify the generalizability of the findings of this study to other types of dispositional characteristics and functioning outcomes.

## 5 Conclusion

These limitations notwithstanding, the present study makes significant contributions to the literature by providing a systematic explanation regarding the effect of dispositional mindfulness on promoting creativity, which supports the perspective of positive psychology that psychological capital/resources are the intermediate pathway through which positive personal characteristics such as dispositional mindfulness can facilitate three aspects of creative functioning including divergent thinking, creative combination, and creative problem solving. Theoretically, these results support the positive psychology perspective by illustrating a positive psychological resource mechanism regarding the positive mindfulness-creativity link. By adopting the multiple-measurement approach to creativity, the results regarding the varied sizes of the mediation effect further enrich the discourse on this perspective by showing that the mediation mechanism may function to different degrees depending on which aspect of creativity is under consideration. Practically, the findings of this study provide evidence of the effectiveness of the current application of mindfulness through the positive effect of a positive psychological state/context facilitated by positive psychological capital/resources. These results can be used as important empirical evidence to support the widespread use of mindfulness in school settings for promoting the positive development of creativity and psychological wellbeing. While mindfulness provides a relatively convenient, economical, and promising way to complement the development of creativity and psychological wellbeing, educators and practitioners may consider adding mindfulness, as a nonjudgmental internal experience-thinking strategy, to training and intervention programs for both creativity and mental health in school settings.

## Data Availability

The datasets presented in this article are not readily available because requests to access the datasets should be directed to the corresponding author, mavishe@eduhk.hk.
